# Comparison of Urine and Genital Samples for Detecting Human Papillomavirus (HPV) in Clinical Patients

**DOI:** 10.1155/2023/7483783

**Published:** 2023-03-27

**Authors:** Hui Yang, Zhao-Yun Luo, Fen Lin, Lie-Jun Li, Min Lu, Long-Xu Xie, Li-Ye Yang

**Affiliations:** ^1^Department of Laboratory Medicine, School of Medicine, Yangtze University, Jingzhou, Hubei 434023, China; ^2^Presicion Medical Center, Chaozhou Central Hospital Affiliated to Southern Medical University, Chaozhou, Guangdong, China; ^3^Chaozhou Hybribio Limited Corporation, Chaozhou, Guangdong, China; ^4^Precision Medical Lab Center, People's Hospital of Yangjiang, Yangjiang 529500, Guangdong, China

## Abstract

**Background:**

Human papillomavirus (HPV) is the main cause of cervical cancer. The aim of the present study was to investigate HPV DNA detection and genotyping on paired genital and urine samples and to evaluate if urine samples could be used to monitor HPV infection.

**Methods:**

Study subjects were recruited from one local hospital in Guangdong of China from September 1, 2011, to June 30, 2012. They were invited to participate if they have taken an HPV genotyping assay for clinical diagnosis of the genital-urinary disease or for a health check-up 3–5 days ago. DNA was extracted from paired genital and urine samples; genotyping was performed with the GenoArray assay.

**Results:**

A total of 250 patients were recruited, which included 203 females and 47 males. Our results showed that the overall agreement on HPV status between the paired samples was 77.1% (155/201, 95% CI: 0.713–0.829) for females, with a kappa value of 0.523 (95% CI: 0.469–0.632), while the agreement was extremely low in the paired male samples. As to individual genotyping, the greatest agreement was found for HPV16 type-specific identification in females (96.02%, 0.933–0.987), followed by the other 12 high oncogenic risk (HR-HPV) types, while the agreement for low-risk HPV detection is poor (*κ* < 0.6). Agreement between paired samples showed that HPV detection had a significantly greater concordance in the samples obtained in females than males (*p* = 0.002). Moreover, the agreement for low-risk HPV detection was significantly lower as compared to HR-HPV detection (48.1% vs. 62.3%, *p* = 0.044).

**Conclusion:**

Despite reduced sensitivity, HPV detection in urine closely represents the same trend that is seen with genital sampling. Urine appears to be an appropriate surrogate sample for HPV DNA detection in women with very limited access to healthcare, while the utility of urine for HPV DNA detection in males is less certain.

## 1. Background

Human papillomavirus (HPV) is one of the most common sexually transmitted infections. It is well established as an etiologic agent in cervical dysplasia and invasive cancer. There are more than 100 different HPV subtypes; approximately 40 HPV subtypes infect the anogenital mucosa. These subtypes can be classified as high-risk, intermediate-risk, and low-risk types, depending on the propensity to cause cervical cancer. At least 15–20 of these subtypes are known to be oncogenic factors [1–3].

HPV-based primary screening or cotesting is being increasingly implemented as an alternative to cytology-based screening to improve cervical cancer prevention and control worldwide. Many different methodologies are available for HPV testing in the setting of cervical cancer screening. HPV testing on gynecologic cytology samples is the most commonly used strategy for cervical cancer screening around the world [4]. However, cervical cancer is still the most common malignancy in women world-wide [3]. This may partly be due to the invasiveness of cytology sampling in the current screening, which is time-consuming and requires a clinician. The use of noninvasive and easy-to-collect samples, such as a self-collected urine sample, may be a useful alternative to cervical specimens for monitoring HPV infection trends, though they do have some significant challenges [5, 6].

Lately, interest in using urine as a liquid biopsy for HPV DNA testing has increased, especially in light of the coronavirus disease 2019 pandemic [7]. High correlations between urine and cervical HPV infections were observed both in developing and developed countries [8–11]. The HPV test using urine appears to be an effective method for detecting HPV infection, though variation is shown in the pooled specificities and sensitivities between urine and the genital detection of HPV across literature [12, 13]. Moreover, most of these studies used samples only from women and few studies in males. To explore evidence in this area, we conduct a study to investigate the concordance of HPV detection and genotyping in paired urine and physician-collected sampling from clinical patients, consisting of both males and females, with the Hybribio GenoArray assay, and to examine if urine samples could be used to monitor HPV infection.

## 2. Materials and Methods

### 2.1. Study Population

This is a cross-sectional study performed at the medical center of Chaozhou Central Hospital, from September 1, 2011, to June 30, 2012. The Ethics Committee of Chaozhou Central Hospital has approved this study. This study was performed in accordance with the Declaration of Helsinki. Most of the study subjects were outpatients, and some from healthy people receiving a routine check-up. They were invited to participate if they have taken an HPV genotyping assay for clinical diagnosis of the genital-urinary disease or for a health check-up 3–5 days before. Additional information, including clinical records and data of cervical liquid-based cytology (LCT) and pathologic examination were reviewed and collected by the ordering physicians from electronic medical records. Eligible participants were those who had no previous treatment for cervical disease (including the loop electrosurgical excision procedure (LEEP), cold knife conization, cryotherapy, and laser therapy), had no previous hysterectomy, had no prior chemotherapy or radiation treatment for cervical neoplasia or another concurrent cancer, had no known HIV infection or AIDS, and were not pregnant at the time of the study.

### 2.2. Sample Collecting and Processing

On the day of their first visit to the hospital, cervical samples were collected from this group of women by the physicians using a cervical brush and were placed in a PCR cell preservation medium (Hybribio Diagnostic China). For men, a clinician-collected sample from the penile shaft, glans penis, coronal sulcus, and scrotum using a saline-soaked swab. After informed consent was obtained, self-collected random urine samples were collected on the day of their second visit to the clinic to get their HPV testing report. All specimens were stored at −20°C and transported to the clinical center of Hybribio Limited Corporation in Chaozhou of China for testing with the clinically validated HPV GenoArray assay.

For the clinical HPV genotyping assay, the cells were precipitated by spinning at 15,000 ×g for 10 min at 4°C for cervical samples and at 4000 ×g for 10 min for genital-based samples according to the manufacturer's instructions. For the urine sample, 10–12 ml of each urine aliquot was pelleted by centrifuged at 4000 ×g for 10 min. After centrifugation, the solid pellets were re-suspended in 200 *μ*l supernatant and were ready for DNA extraction using the alkaline lysis-based method kit supplied with the HPV GenoArray assay (Hybribio Biotechnology Limited Corp.).

### 2.3. HPV GenoArray Assay

Genotyping for HPV was then done by DNA amplification, flow-through hybridization, and GenoArray assay by HybriMax (Chaozhou Hybribio Limited Corp., Chaozhou, China). The test was performed according to the manufacturer's instructions. Detailed protocols for this assay had been described previously [13, 14]. The GenoArray assay could identify 13 HR-HPVs (16, 18, 31, 33, 35, 39, 45, 51, 52, 56, 58, 59, and 68), five low-risk HPVs (LR-HPVs) (6, 11, 42, 43, and 44), and three popular HPV type 53, 66, and 81 (CP8304) in the Chinese population.

### 2.4. Statistical Analysis

Agreement for HPV DNA detection in paired urine-genital specimens was evaluated using the kappa coefficient (*κ*), and its corresponding 95% CI. Kappa values below 0.2 were considered as poor agreement, kappa values between 0.21 and 0.40 were considered as fair, while values between 0.41 and 0.60 as moderate and values between 0.61 and 0.80 as substantial agreement, and kappa values greater than 0.81 were considered as almost perfect agreement. Among HPV positive samples, interspecies genotype agreement with the genotype assay was also determined. For this comparison, genotype results were categorized as: (i) HPV16/18, (ii) other high-risk HPV types, including HPV31, −33, −35, −39, −45, −51, −52, −56, −58, −59, −66, and/or −68, (iii) HPV6/11, and (iv) three other low-risk HPVs (42, 43, and 44) and three popular HPV types reported in the Chinese population.

## 3. Results

A total of 250 patients were recruited, which included 203 females and 47 males. After excluding 11 subjects because of the low DNA quality of their urine samples (a negative result for beta-globin of internal control), 190 (154 females and 36 males) clinically tested HPV positive and 49 (2 males and 47 females) clinically tested HPV negative subjects were included in the final statistical analysis. The median age of the 239 eligible participants was 33 years (range 17–65). Fifty-six of this population had multiple infections with the cervix-genital samples. Cytology and histological data of cervix-genital samples from the participants were retrieved from the clinical records. A total of 195 female participants originally underwent cytology and HPV cotesting of cervical samples (148 with HPV testing positive and 47 with HPV negative). Of these HPV testing positive female cases, 137 (92.6%) had normal cytology, 10 (6.8%) had a borderline or mild dyskaryosis reading (i.e., 1 with AGC (atypical glandular cells), 1 with ASC-US (atypical squamous cells of undetermined significance), 1 with ASC-H (atypical squamous cells, cannot rule out a high-grade lesion), 7 with LSIL (low-grade intraepithelial lesion)), and 1 (0.7%) had HSIL histologically confirmed with CIN 3. All the remaining 47 females with a negative HPV testing had normal cytology.

The carcinogenic HPV test results in both sample sources were presented according to the cervical cytology in Table 1. The relatively high prevalence of HPV infection and the low prevalence of precancerous lesions in this study population did not allow to reliably evaluating the sensitivity and specificity of HPV detection from a urine sample for cervical cancer screening. Anyway, the vast majorities of women with abnormal cytology findings (10/11) tested positive for high-risk HPV infection in both sample sources.

**Figure 1:**
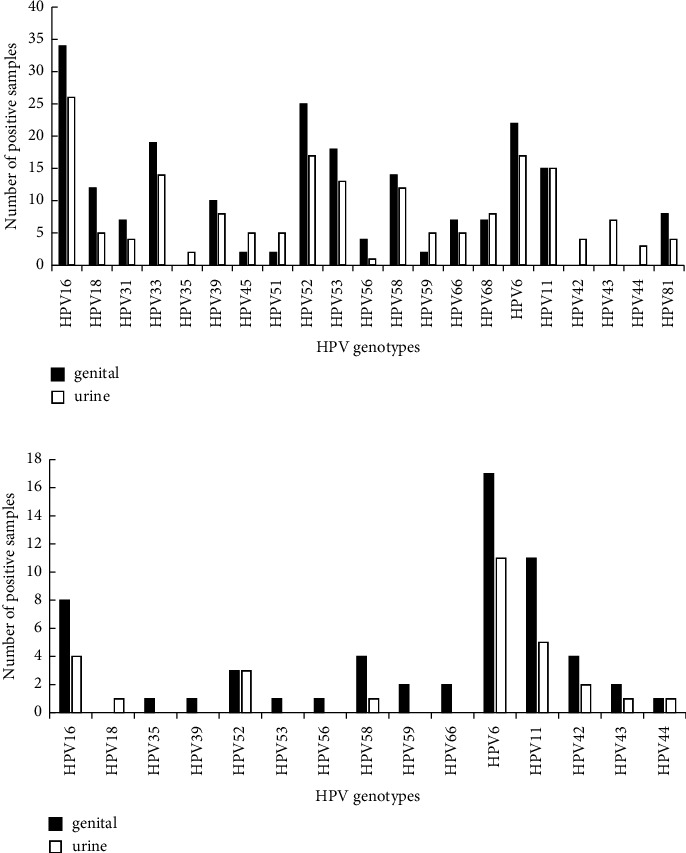
HPV genotype distribution in genital (black bars) and urine (white bars) samples from both (a) females (*n* = 154) and (b) males (*n* = 36).

HPV detection and genotype distribution from each source sample (cervix/genital samples and urine samples) for the 239 eligible participants are detailed in Table 2. Males and females were separately analyzed. For females, the genotyping results showed that HPV16 was the most prevalent genotype in both cervix and urine samples, and HPV52 was the second prevalent genotype detected in the cervical sample, while in urine sample, both HPV52 and HPV6 were the second prevalent (Figure 1(a)). For males, the most frequent HPV type was HPV6 from each source sample (genital and urine samples), followed by HPV11 and HPV16 (Figure 1(b)).

**Figure 2:**
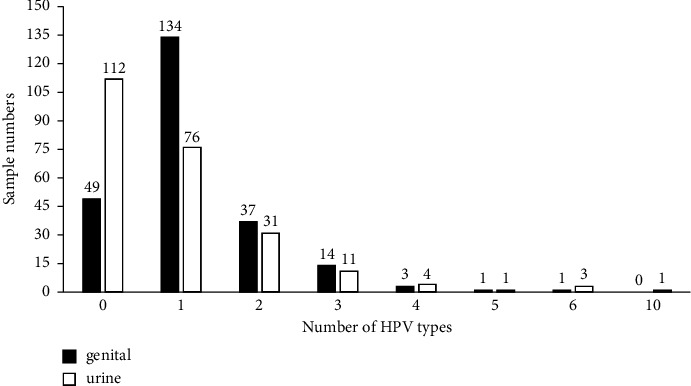
Status of multiple HPV infection in 239 paired genital and urine samples.

23.4% (*n* = 56; 95% CI: 0.180–0.288) of the genital samples were positive for at least two subtypes, compared to 21.3% (*n* = 51; 95% CI: 0.161–0.265) of the urine samples. Such a difference does not reach statistically significant levels (*x*^2^ = 0.301; *p* = 0.58) for coinfection detection (Figure 2).

The results of HPV status and type-specific prevalence in urine samples were compared to those detected in clinical samples. The overall agreement on HPV status between the paired samples was 77.1% (155/201, 95% CI: 0.713–0.829) for females, with the kappa value of 0.523 (95% CI: 0.418–0.628) and 52.6% (20/38, 95% CI: 0.367–0.685) for males, with the kappa value of 0.095 (95% CI: 0–0.224). The remaining 64 cases (18 males and 46 females) with discrepant HPV results were all negative in the urine sample but positive in the genital sample.

With respect to individual genotyping, considering only the 21 HPV subtypes present in clinical assays, the agreement for detection of HPV16/18 between urine and clinician-collected genital-reproductive samples were 91.5% (184/201, 95% CI: 0.876–0.954) in females, with the kappa value of 0.729 (95% CI: 0.610–0.848). While in males, agreement for detection of HPV16/18 was 89.5% (34/38, 95% CI: 0.798–0.992), with the kappa value of 0.612 (95% CI: 0.282–0.942). For poor detection of 12 high-risk HPV genotypes other than HPV16 and HPV18, the overall agreement was 81.6% (31/38, 0.693–0.939) in males and 80.1% (161/201, 0.746–0.856)in females between matching samples, *κ* values were of 0.424 (0–0.771) and 0.57(0.455–0.685) in males and females, respectively (Table 3).

As for the 5 low-risk HPV (HPV6, HPV11, HPV42, HPV43, and HPV44) and other 3 popular HPVs (HPV53, HPV66, and HPV81) detection, the overall concordance of the paired samples was slight, *κ* value was 0.458 (0.310–0.606) in females and 0.058 in males. While for 6/11 alone, the agreement could reach 87.56% (176/201, 0.83–0.92) in female samples, with *κ* value of 0.552 (95% CI: 0.398–0.706) (Table 3).

**Figure 3:**
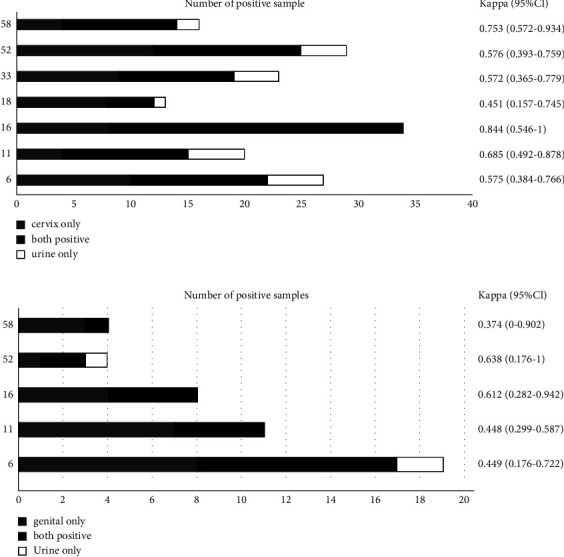
HPV genotype-specific detection differences between genital and urine samples from (a) females and (b) males. Bars indicate the number of samples positive in both samples (black bars), genital samples only (gray bars), or urine only (white bars).

Agreement between paired samples showed that HPV DNA detection had a significantly greater concordance in the samples obtained in females than males (*p* = 0.002). Moreover, the greatest agreement was found for HPV16 for type-specific identification in females (96.02%, 0.933–0.987), followed by the other 12 HR-HPV identification, while the agreement for low-risk HPV detection was significantly lower as compared to HR-HPV detection (48.1% vs. 62.3%, *p* = 0.044) (Figure 3).

## 4. Discussion

Increasing studies have evaluated urine-based sampling for HPV DNA detection in comparison with cervical or other external genital sampling in clinical settings, but the outcomes indicated variations between the pooled specificities and sensitivities. Consistent with most of the previous studies [15–17], the results from the present study confirm that, HPV detection in urine samples in females is possible, though the detection sensitivity is significantly lower than that in paired cervical samples. In addition, according to the data obtained in the present study, urine samples, especially random urine samples do not seem to be optimal sampling for monitoring HPV prevalence in males due to the significantly reduced sensitivity in the urine sample. Payan et al. [18] reported a marked difference of HPV viral load across the urine and cervical samples. Therefore, we inferred that the reduced sensitivity in urine sample may be caused by significantly lower exfoliated cells in the male urine sample.

A limitation of the present study was that the urine samples used were random urine, not the first void urine [19]. This is one possible explanation that we only found an overall agreement of 77.1% and a moderate concordance rate of HPV DNA detection in paired urine and cervical samples. The results obtained in this study were similar to those obtained in Sahasrabuddhe et al. [20] and Keimari Mendez et al. [9] studies in outpatients and Munoz et al., [21] study in HIV-infected women. The agreement of random urine is lower than that in paired first-void urine and cervical samples [17, 18].

In this study, we demonstrated a uniform genotype distribution across genital and urine sampling methods. For instance, HPV genotyping from both genital and urine samples showed that HPV16 were the most frequent HPV type found in females, while in males the commonest genotype was HPV6. Although the frequency of HPV infection was lower in the urine samples than in the genital sample, the differences in detection rates across sampling approaches were not statistically significant. We also found that the low-oncogenic risk HPV type had a higher prevalence in male genital and urine specimens than in females. These results were similar to the previous epidemiology studies of human papillomavirus infection from genital samples in the same region [22, 23].

As expected, we found a higher concordance rate between urine and genital samples for HPV16 and any of other HR-genotype detection than for low oncogenic risk HPV genotype, especially in males. This result was consistent with other published studies [8, 12, 15, 20, 24]. It has been reported that high-risk HPV infection may cause more exfoliated cervical cells in urine, and reflected a higher grade of cervical lesions compared with those low oncogenic risk HPV. The results obtained in this study may support the use of urine as appropriate surrogate for HPV screening and genotyping in women with more severe cervical disease. Additional studies are necessary to evaluate the performance of HPV testing using urine in those high-risk populations.

In conclusion, HPV detection in urine closely represents the same trend as for genital sampling, although the detection sensitivity in the urine specimen is significantly reduced, urine sampling appears to be a suitable surrogate sample for HPV type-specific detection in women with very limited access to healthcare, while the utility of urine for HPV DNA testing in males is less certain. Further study on the improvement of the HPV DNA detection rate in the urine sample is still needed.

## Figures and Tables

**Table 1:** Detection of carcinogenic HPV types in urine samples and cervix samples according to the cytological results.

Cytology diagnosis	No.(%) of women with HR-HPV detection in paired cervix and urine sample				*κ* (95% CI)
	Both positive	Genital + urine −	Genital − urine +	Both negative	
Abnormal (*n* = 11)	10	1	0	0	NA
Normal (*n* = 185)	70	37	7	70	0.53 (0.46–0.65)

**Table 2:** HPV detection and type-specific distribution from each source sample (genital and urine) in male and female patients with genital-urinary disease.

	Female (*n* = 201)				Male (*n* = 38)			
	Both positive	Genital sample	Urine sample	Both negative	Both positive	Genital sample	Urine sample	Both negative
		Only	Only			Only	Only	
HPV16	26	8	0	167	4	4	0	30
HPV18	4	8	1	188	0	0	1	37
HPV31	4	3	0	194	0	0	0	38
HPV33	10	9	4	178	0	0	0	38
HPV35	0	0	2	199	0	1	0	37
HPV39	5	5	3	188	0	1	0	37
HPV45	2	0	3	196	0	0	0	37
HPV51	1	1	4	195	0	0	0	37
HPV52	13	12	4	172	2	1	1	34
HPV56	0	4	1	196	0	1	1	36
HPV58	10	4	2	185	1	3	0	34
HPV59	1	1	4	195	0	2	0	36
HPV68	4	3	4	190	0	0	0	38
HPV6	12	10	5	174	9	8	2	19
HPV11	11	4	5	181	4	7	0	27
HPV42	0	0	4	197	1	2	1	34
HPV43	0	2	5	194	0	1	1	36
HPV44	0	2	1	198	0	0	0	38
HPV53	10	8	3	180	0	1	0	37
HPV66	4	3	1	193	1	1	0	36
HPV81	3	5	1	192	0	2	0	36

**Table 3:** Agreement between urine samples and genital samples for HPV detection and genotyping.

Study	HPV types	No. of paired genital and urine sample				*κ* (95% CI)
		Both positive	Genital + urine −	Genital − urine +	Both negative	
Female (*N* = 201)	HR-HPV	74	41	8	78	0.53 (0.416–0.634)
	16/18	30	16	1	154	0.73 (0.610–0.848)
	Other HR	52	28	12	109	0.57 (0.455–0.685)
	LR-HPV^∗^	25	21	16	139	0.46 (0.310–0.606)
	6/11	21	14	11	155	0.55 (0.398–0.706)

Male (*N* = 38)	HR	7	8	1	22	0.46 (0.186–0.736)
	16/18	4	4	0	30	0.61 (0.282–0.942)
	Other HR	4	5	2	27	0.42 (0.077–0.771)
	LR-HPV^∗^	12	20	3	3	−0.058 (0–0.146)
	6/11	13	14	1	10	0.29 (0.065–0.513)

^∗^LR-HPV included HPV6, 11, 42, 43, 44, 53, 66, and 81.

## Data Availability

The datasets generated and/or analyzed during the current study are not publicly available due to the regulation of Chaozhou Central Hospital, but are available from the corresponding author upon request.
